# Production of omega-3 enriched tilapia through the dietary use of algae meal or fish oil: Improved nutrient value of fillet and offal

**DOI:** 10.1371/journal.pone.0194241

**Published:** 2018-04-11

**Authors:** Tyler R. Stoneham, David D. Kuhn, Daniel P. Taylor, Andrew P. Neilson, Stephen A. Smith, Delbert M. Gatlin, Hyun Sik S. Chu, Sean F. O’Keefe

**Affiliations:** 1 Department of Food Science and Technology, Virginia Polytechnic Institute and State University, Blacksburg, Virginia, United States of America; 2 Department of Biomedical Sciences and Pathology, Virginia-Maryland Regional College of Veterinary Medicine, Blacksburg, Virginia, United States of America; 3 Department of Fisheries and Wildlife Sciences, Texas A&M University, College Station, Texas, United States of America; University of Illinois, UNITED STATES

## Abstract

The goal of this project was to increase the nutrient value of fillets, by-product muscle, and offal of aquacultured tilapia. A diet that includes seafood with a high omega-3 (n-3) fatty acid content, more specifically eicosapentaenoic acid (EPA) and docosahexaenoic acid (DHA), are known to have numerous health benefits for consumers. Improved nutrient value of the offal may also attract new market opportunities for the aquaculture industry. Tilapia were cultured on different experimental feeds that contained various levels of n-3 fatty acids from either fish oil (FO) or algae meal (AM) that were used to replace corn oil. The experimental diets included a control (corn oil 6.3%), FO1%, FO3%, FO5%, AM1.75%, AM5.26%, and AM8.77%. All diets were formulated to be isocaloric, isonitrogenous, and isolipid. Three hundred and fifty tilapia with an initial mean weight of 158±2 g were cultured in a recirculating aquaculture system (seven diets replicated at the tank level, 14 tanks, 25 fish per tank). For all of the production performance data, no differences (P>0.05) were observed between the experimental groups which included survival (overall mean ± standard error, 99.4±0.3%), growth per week (45.4±1.0 g/wk), food conversion ratio (1.32±0.03), fillet yield (44.4±0.2%), hepatosomatic index (1.61±0.02), viscerosomatic index (2.86±0.06), and mesenteric fat index (0.97±0.04). Fillet and rib meat tissues were collected at weeks four and eight, and liver and mesenteric fat tissues were collected at week eight. Fatty acids were extracted, methylated and identified with gas chromatography–mass spectrometry. All tissues had improved fatty acid profiles (higher n-3, lower n-6, n-6:n-3) with increasing levels of FO and AM in the diet. For example, the best diet for significantly (P<0.05) improving the lipid profile in tilapia fillets at week eight was diet AM8.77%. In the fillet, total n-3 was increased (control versus AM8.77%) from 151.2±19.0 to 438.7±14.2 mg per 4 ounce (113 g) serving and n-6:n-3 ratio was improved from 5.19±0.76 to 1.29±0.03.

## Introduction

Tilapia is a healthy food choice for consumers because it is a relatively low-fat fish that is rich in proteins and minerals. Tilapia is the second most cultivated freshwater fish worldwide, typically yielding between 30–40% fillet yield leaving 60–70% processing waste commonly referred as offal [1, 2]. This has often led to relatively low margins for tilapia fillets compared to other finfish species [1]. However, there is an opportunity for producers to further improve the nutrient value (e.g. healthy fats) of tilapia fillets and offal through manipulations of tilapia feed leading to higher value products in the market place.

The benefits of healthy fats, omega-3 (n-3) fatty acids, to humans include prevention of cardiovascular disease, improvement of visual acuity, and fortification of mental health. For this reason the American Heart Association (AHA) recommends two 4 oz (113 g) servings of fatty fish that are high in omega-3 fats (i.e. salmon) per week [3]. Omega-3 fatty acids include, among others, alpha linolenic acid (ALA 18:3 n-3), eicosapentaenoic acid (EPA 20:5 n-3), docosapentaenoic acid (DPA 22:5 n-3) and docosahexaenoic acid (DHA 22:6 n-3). However, not all n-3 fatty acids are equally beneficial to humans [4]. Due to the low efficiency of converting ALA into longer chain n-3 fatty acids (<10%), ALA is of relatively little benefit to humans [5]. Meanwhile, long-chain polyunsaturated fatty acids (LC-PUFAs); EPA, DPA, and DHA are significantly more beneficial to human health and development. Conversely, diets high in n-6 fats (high dietary n-6:n-3 ratios) lead to human health deficits including inflammation, asthma and reduced kidney function [6].

Fish oil (FO) and microalgae has been found to be a possible feed ingredient for enriching LC-PUFAs in channel catfish, Atlantic salmon, and seabream [7–10]. In general, attempts to enrich LC-PUFAs in tilapia fillets using plant oil alternatives have been relatively unsuccessful. Diets supplemented with flaxseed have been found to increase ALA and LC-PUFAs significantly (P<0.05) in liver, but, not significantly in tilapia fillets [11, 12]. Compared to macroalgae, microalgae has less fiber and is generally higher in lipid content [13]. Recently, microalgae (*Schizochytrium* sp.) was successfully used in fish diets to improve production characteristics and the fatty acid profile in young tilapia (approximate mean weight of 25 g) [14]. Moreover, all of the aforementioned studies aimed to enhance n-3 fatty acids in fish fillets, not in the other tissues (e.g. offal).

The goal of the study herein was to evaluate if diets supplemented with FO and algae meal (AM) can provide an enrichment of LC-PUFAs and reduction of n-6:n-3 ratio in fillets and offal (including rib meat, liver, and mesenteric fat) of market size fish (greater than 500 gram fish).

## Materials and methods

### Fish and culture system

All procedures have been approved by Virginia Tech’s Institute of Animal Care and Use Committee (VT-IACUC-#14–211). Juvenile tilapia (*Oreochromis niloticus*, ~11 grams each*)* were shipped from Spring Genetics (Akvaforsk Genetics Center, Miami, Florida, U.S.) to Virginia Tech’s aquaculture facilities (Blacksburg, Virginia, U.S.). Fish were acclimated and conditioned for 4 weeks until they reached a mean individual size of approximately 160 grams prior to experiment initiation. Fish were cultured in an indoor recirculating aquaculture systems (RAS) equipped with fourteen 1-meter-diameter polyethylene tanks (~250 liters each), bubble-bead filters for mechanical filtration, fluidized-bed bioreactors for biological treatment, UV disinfection units, heat exchangers, and distributed diffuse aeration.

Water quality in the RAS was rigorously monitored throughout the nutritional study. All water quality parameters were analyzed using methods adapted from APHA [15]. Dissolved oxygen and temperature were monitored daily. Alkalinity, total ammonia nitrogen (TAN), nitrite, nitrate and pH values were analyzed three times a week.

### Diets

The FO and AM used in this study was Virginia Prime-Gold® (Omega Protein, Houston, Texas, U.S.) and *Schizochytrium* sp. (Alltech, Nicholasville, Kentucky, U.S.). The fatty acid profile of both lipid sources are presented in Table 1. Because algae was in meal form instead of oil additional proximate data was collected. Proximate data for algae meal was 18.8, 3.70, 3.67, and 24.9% protein, moisture, ash, and carbohydrates, respectively. All experimental diets were formulated on an isonitrogenous and isocaloric basis. The pelleted experimental feed formulations are presented in Table 2. The independent variable for this experiment was the lipid composition of the seven diets. Dependent variables were survival rate, growth, biometrics, performance indices, feed conversion ratio (FCR), and nutritionally relevant fatty acids.

**Table 1:** Composition of fatty acids (%) in fish oil and lipid fraction of algae meal.

	Fish oil	Algae meal
14:0	8.04	3.86
16:0	16.85	54.69
18:0	3.09	1.8
16:1 n-7	11.5	0
18:1 n-9	9.74	0
18:2 n-6	1.89	0
20:4 n-6	2.49	0
22:1 n-9	0	0.53
22:2 n-6	0	0.43
18:3 n-3	2.2	0
20:5 n-3	14.05	0.37
22:5 n-3	2.95	0
22:6 n-3	12.26	27.2

**Table 2:** Composition of experimental feeds (g/100 g (%) as-is basis).

	Control	Fish oil			Algae meal		
		1%	3%	5%	1.75%	5.26%	8.77%
Soybean (46.5%)^1^	37.0	37.0	37.0	37.0	37.0	37.0	37.0
Wheat^2^	37.7	37.7	37.7	37.7	37.5	37.2	37.0
Meat and bone meal^3^	13.8	13.8	13.8	13.8	13.4	12.1	10.9
Fishmeal (menhaden)^4^	5.0	5.0	5.0	5.0	5.0	5.0	5.0
Corn oil^5^	6.3	5.3	3.3	1.3	5.1	3.2	1.1
Fish oil^4^	0	1.0	3.0	5.0	0	0	0
Algae meal^6^	0	0	0	0	1.75	5.26	8.77
Vitamin premix^7^	0.1	0.1	0.1	0.1	0.1	0.1	0.1
Mineral premix^7^	0.1	0.1	0.1	0.1	0.1	0.1	0.1

^1^ADM Alliance Nutrition

^2^Southern States Cooperative, Richmond, Virginia, US

^3^Smithfield—Farmland, Smithfield, Virginia, US

^4^Omega Protein, Houston, Texas, US

^5^Kroger, Cincinnati, Ohio, US

^6^Alltech, Nicholasville, Kentucky, US

^7^Purina, St. Louis, Missouri, US

All diets were analyzed to confirm their proximate nutritional values (Table 3) and essential amino acids (Table 4) using a commercial lab (Midwest Labs, Omaha, NE, USA). Fatty acid profiles for each diet are reported in Table 5. Feeding rates were determined for all treatment groups on a percent of body weight per day basis. Monitoring the amount of feed consumed allowed FCR to be determined. Tilapia were group-weighed on a per tank basis weekly to enable appropriate feed adjustments. Feeding rates were 4.0, 3.75, 3.25, 3.25, 2.375, 2.0, and 1.85 percent body weight per day (% BW/d) for weeks 1, 2, 3, 4, 5, 6, 7 and 8, respectively.

**Table 3:** Determined energy, nutrients, and trace element levels in various treatment diets (dry-matter basis).

		Fish oil			Algae meal		
Parameter	Control	1%	3%	5%	1.75%	5.26%	8.77%
**Caloric content (cal/g)**							
Total calories	4940	4830	4810	4930	4900	4880	4850
**Proximate and mineral levels**							
Crude protein	37.0	36.5	35.5	35.7	37.3	35.5	36.5
Carbohydrate^1^	33.7	36.6	37.7	37.3	36.4	37.9	35.7
Total ash	9.18	9.19	9.12	8.96	8.80	8.55	8.38
Crude fat	10.20	9.17	9.55	9.56	10.20	10.30	10.60
Crude fiber	4.00	4.50	4.00	3.80	5.10	3.90	5.80
Calcium	2.08	2.14	2.11	2.10	2.10	2.01	1.91
Phosphorus	1.41	1.45	1.42	1.40	1.40	1.33	1.27
Potassium	1.31	1.25	1.28	1.29	1.30	1.26	1.29
Magnesium	0.24	0.23	0.24	0.24	0.24	0.27	0.26
Sodium	0.16	0.15	0.15	0.16	0.14	0.16	0.15
**Trace element levels (ppm)**							
Iron	226	215	224	223	222	224	208
Copper	24	21	22	21	21	21	21
Zinc	305	285	317	272	274	299	271
Manganese	101	71	88	122	101	83	92

^1^Calculated value (Merrill and Watt, 1973): Carbohydrate = total—(ash + crude protein + moisture + total fat)

**Table 4:** Determined essential amino acids in various treatment diets (dry-matter basis).

	Control	Fish oil			Algae meal		
		1%	3%	5%	1.75%	5.25%	8.77%
Arginine^1^	2.57	2.48	2.46	2.47	2.35	2.47	2.43
Histidine^2^	0.84	0.81	0.77	0.83	0.84	0.81	0.78
Isoleucine^3^	1.45	1.22	1.32	1.44	1.25	1.40	1.24
Leucine^4^	2.38	2.30	2.27	2.41	2.38	2.30	2.25
Lysine^5^	1.99	1.90	1.89	2.01	2.00	1.89	1.92
Methionine^6^	0.49	0.46	0.48	0.48	0.42	0.41	0.44
Phenylalanine^7^	1.61	1.55	1.56	1.62	1.58	1.49	1.54
Threonine^8^	1.36	1.35	1.29	1.33	1.39	1.25	1.36
Tryptophan^9^	0.35	0.25	0.23	0.31	0.30	0.27	0.26
Valine^10^	1.64	1.68	1.61	1.81	1.65	1.77	1.42

^1^(204)Method: AOAC 994.12 (Alt. III) total amino acids-hydrolysis Units: %Basis

^2^(209)Method: AOAC 994.12 (Alt. III) total amino acids-hydrolysis Units: %Basis

^3^(210)Method: AOAC 994.12 (Alt. III) total amino acids-hydrolysis Units: %Basis

^4^(211)Method: AOAC 994.12 (Alt. III) total amino acids-hydrolysis Units: %Basis

^5^(total) (195)Method: AOAC 994.12 (Alt. III) total amino acids-hydrolysis Units: %Basis

^6^(212)Method: AOAC 994.12 (Alt. I)—cystine & methionine Units: %Basis

^7^(213)Method: AOAC 994.12 (Alt. III) total amino acids-hydrolysis Units: %Basis

^8^(217)Method: AOAC 994.12 (Alt. III) total amino acids-hydrolysis Units: %Basis

^9^(218)Method: AOAC 988.15—tryptophan Units: %Basis

^10^(220)Method: AOAC 994.12 (Alt. III) total amino acids-hydrolysis Units: %Basis

**Table 5:** Determined fatty acid levels in various treatment diets (dry-matter basis).

	Control	Fish oil			Algae Meal		
Fatty acid		1%	3%	5%	1.75%	5.26%	8.77%
**Total SFA**	**23.22**	**26.00**	**29.34**	**35.22**	**28.08**	**39.95**	**46.78**
12:0	0	0.04	0.08	0.1	0	0.08	0.13
14:0	0.78	2.26	4.28	6.54	1.45	2.99	4.45
15:0 ANTEISO	0	0.2	0.39	0.56	0.28	0.81	1.24
15:0 ISO	0	0.06	0.14	0.21	0	0	0
16:0	17.07	16.30	17.16	20.76	21.75	30.60	36.18
17:0	0.10	0.25	0.44	0.49	0	0.34	0.43
18:0	4.88	6.36	6.43	6.28	4.30	4.77	4.08
20:0	0.39	0.53	0.42	0.28	0.30	0.36	0.27
**Total MUFA**	**31.66**	**32.37**	**32.12**	**32.13**	**28.92**	**20.48**	**14.85**
14:1 n-5	0	0	0	0	0	0	0.11
16:1 n-11	0	0	0	0	0	0	0
16:1 n-9	0	0.19	0.13	0.19	0	0	0
16:1 n-7	1.05	3.12	5.90	8.88	0.98	0.93	0.96
18:1 n-9	28.77	26.04	22.4	19.04	26.41	18.26	12.72
18:1 n-7	1.44	2.35	2.91	3.23	1.21	1.03	0.90
20:1 n-11	0	0	0	0	0	0	0
20:1 n-9	0.40	0.67	0.78	0.79	0.32	0.26	0.16
**Total PUFA**	**45.12**	**41.62**	**38.55**	**32.65**	**43.01**	**39.58**	**38.37**
18:2 n-6	43.53	36.47	29.41	21.1	38.91	26.73	16.82
18:3 n-6	0	0.05	0.13	0.15	0	0	0
18:3 n-3	1.59	2.09	2.12	1.93	1.07	1.06	0.88
20:2 n-6	0	0.18	0.22	0.20	0	0	0
20:3 n-3	0	0	0.11	0	0	0	0
20:5 n-3	0	0.40	0.70	0.92	0	0	0
22:4 n-3	0	0	0	0	0	0	0
22:5 n-6	0	0	0	0	0	2.23	4.00
22:5 n-3	0	0.42	1.08	1.43	0	0	0
22:6 n-3	0	2.01	4.78	6.92	3.03	9.56	16.67
**% Omega 3**	**1.59**	**4.92**	**8.79**	**11.20**	**4.10**	**10.62**	**17.55**
**% Omega 6**	**43.53**	**36.70**	**29.76**	**21.45**	**38.91**	**28.96**	**20.82**
**Omega 6:3**	**27.38**	**7.46**	**3.39**	**1.92**	**9.49**	**2.73**	**1.19**
**% Beneficial Omega-3**	**0**	**2.83**	**6.56**	**9.27**	**3.03**	**9.56**	**16.67**

SFA:Saturated fatty acids, MUFA:Monounsaturated fatty acids, PUFA:Polyunsaturated fatty acids. LC-PUFAs includes EPA, DPA and DHA.

### Feed management

Feed was stored in a commercial refrigerator at a temperature between 0 and 3.5°C until it was used. Feed rates were consistent between all treatment groups on a percent body weight per day basis. Tilapia were weighed weekly on a per tank basis to adjust feed amounts based on weight gains. Growth and the corresponding feed amount was projected each week to account for projected daily growth. Feed was loaded on a twenty-four hour belt feeder to deliver feed hourly over an 18 hour period.

### Biometrics

Fillet yield, hepatosomatic index (HSI), viscerosomatic index (VSI), and mesenteric fat index (MFI) were determined by dividing the fillet/muscle tissue, liver, total viscera mass, and mesenteric/visceral fat by the whole weight of the fish, respectively.

### Tissue sampling

Fillet and rib meat tissues were collected at weeks four and eight, and liver and mesenteric fat tissues were collected at week eight. Rib meat for the purposes of this study is comprised of the pin bones and belly meat ventral to the fillet. Tissue samples were collected on a per tank/diet basis with two samples for each tissue originating from each tank. Each of these two samples were a pooled sample containing tissues from two fish of the same treatment. Samples were vacuum packed with 10 ml of methanol in order to deactivate enzymes and then quick-frozen in a bath of isopropanol and dry ice. These samples were then stored at −80°C until analysis.

Lipid extraction was performed according to Bligh and Dyer [16] and methyl esters were prepared and analyzed according to Ackman [17] and AOCS [18]. The AOCS [18] method Ce-1b-89 was used with a QP-2020 Ultra Gas Chromatography–Mass Spectrometry (GC-MS) (Shimadzu Corp., Kyoto, Japan) to determine the fatty acid profiles of each sample. Methylation following the AOCS [18] Ce-1b-89 procedure was followed by GC-MS using a QP-2020 fitted with a flexible fused silica wall coated open tubular column (Zebron 60m x 0.25mm i.d., 0.25 um film thickness) operated using helium carrier gas at 25 cm/sec linear flow velocity. The split ratio was 1:50, the injection port held at 250°C and transfer line held at 220°C. Fatty acid methyl esters were identified based on mass spectra and ECL values that were calculated according to Ackman [17]. Since DHA, DPA and EPA are of the greatest benefit to humans, those fatty acids have been combined and defined as “beneficial n-3 fatty acids” or LC-PUFAs.

### Data analysis

Statistical analysis was performed using JMP Pro 11 for Apple (Cary, NC, USA). One-way ANOVA was utilized when more than two means were compared to determine dietary effects on dependent variables (fish performance, biometrics, tissue fatty acid composition). When appropriate, Tukey’s post-hoc test was applied to determine where the significant (P < 0.05) differences occurred amongst the means.

## Results

Water quality averages were: temperature 29.4°C, pH 7.79, dissolved oxygen 5.45 mg/L, alkalinity 199 mg/L, total ammonia-N 0.35 mg/L, nitrite-N 0.06 mg/L, and nitrate-N 11.8 mg/L in the RAS over the experimental period. These conditions are considered optimal for tilapia culture [1, 19]. Nutritional profiles were consistent across each of the experimental diets (Tables 3–5). Meanwhile, no significant differences were observed between fat content of the various tissues of fish fed the different diets (S1 Table).

Fish performance and biometric results are presented in Table 6. No significant differences between survival, growth, FCR, or any biometrics were observed for fish fed the different experimental diets.

**Table 6:** Effects of diets on fish growth and biometrics.

	Control	Fish oil			Algae meal			P
		1%	3%	5%	1.75%	5.26%	8.77%	
**Tilapia performance**								
Survival (%)	98.0 ± 1.4	100 ± 0.0	98.0 ± 1.4	100 ± 0.0	100 ± 0.0	100 ± 0.0	100 ± 0.0	0.2020
Initial weight (g)	161.4 ± 0.4	156.9 ± 1.3	156 ± 1.1	158.1 ± 0.4	161.7 ± 1.1	154.6 ± 2.2	156.6 ± 0.9	0.1344
4 week weight (g)	330.6 ± 3.0	315.9 ± 7.1	315.6 ± 1.4	335.0 ± 0.2	333.0 ± 4.2	328.8 ± 18.0	310.5 ± 9.2	0.8554
8 week weight (g)	521.3 ± 12.5	504.5 ± 7.3	513.4 ± 10.5	539.7 ± 12.7	561.7 ± 3.2	521.3 ± 45.2	484.0 ± 18.5	0.4356
Average weight gain (g/Week)	45.0 ± 1.6	43.4 ± 0.9	44.7 ± 1.3	47.7 ± 1.6	50.0 ± 0.4	45.8 ± 5.6	40.9 ± 2.3	0.5214
FCR	1.46 ± 0.01	1.31 ± 0.01	1.31 ± 0.0	1.24 ± 0.03	1.26 ± 0.05	1.30 ± 0.12	1.37 ± 0.06	0.3982
**Biometrics at 8 weeks**								
Fillet yield	45.3 ± 0.6	45.0 ± 0.5	43.8 ± 0.6	44.3 ± 0.6	43.9 ± 0.7	44.6 ± 0.6	43.6 ± 0.7	0.1467
Hepatosomatic index	1.60 ± 0.06	1.52 ± 0.08	1.61 ± 0.09	1.59 ± 0.13	1.61 ± 0.07	1.65 ± 0.09	1.71 ± 0.10	0.5218
Viscerasomatic index	3.04 ± 0.28	2.83 ± 0.29	2.97 ± 0.20	2.61 ± 0.24	2.64 ± 0.33	3.06 ± 0.24	2.90 ± 0.14	0.7490
Mesenteric fat index	0.87 ± 0.06	0.98 ± 0.10	1.03 ± 0.18	1.05 ± 0.07	1.17 ± 0.14	0.85 ± 0.15	0.87 ± 0.13	0.6473

Mean values with standard errors and one-way ANOVA statistics.

Week four fillet and rib meat fatty acid data are presented in Table 7. Significant differences (P<0.05) were observed for ALA, DHA, n-6, n-6:n-3, and LC-PUFAs in the fillet and rib meat (with the addition of EPA) of the fish fed different diets. The best diet of the fish oil diets, FO5%, resulted in an increase of n-3 and LC-PUFAs of 41 and 76%, respectively, compared to the control group. With a corresponding decrease in n-6 and n-6:n-3 by 36 and 55%, respectively. The best diet in regards to improving the fatty acid profile was AM8.77% resulting in a n-3 and LC-PUFAs increase of 96 and 163% over the control diet, respectively. Meanwhile, n-6 and n-6:n-3 ratio were decreased by 37% and 67%. Fatty profile changes were similar for the rib meat. The major difference between rib mean and fillets at four weeks was the rib meat contained twice as much crude fat.

**Table 7:** Nutritional fatty acid profiles of tilapia fillet and rib meat after 4 weeks of dietary treatments.

	Control		Fish oil						Algae meal						
		^ ^	1%	^ ^	3%	^ ^	5%	^ ^	1.75%	^ ^	5.26%	^ ^	8.77%	^ ^	P
**Fillet**															
mg ALA	23.3 ± 0.9	^a,b^	23.7 ± 0.7	^a,b^	16.1 ± 1.7	^c^	21.4 ± 0.8	^a,b^	25.9 ± 0.4	^a^	16.0 ± 0.8	^c^	19.4 ± 0.2	^b,c^	<0.0001
mg EPA	6.4 ± 1.1		16.9 ± 6.6		12.6 ± 1.6		18.9 ± 6.1		5.5 ± 0.5		4.2 ± 0.6		7.2 ± 0.4		0.0894
mg DPA	19.3 ± 4.1		42.9 ± 14.4		34.2 ± 4.7		42.0 ± 10.1		16.0 ± 1.4		10.7 ± 0.6		16.5 ± 0.5		0.0502
mg DHA	64.8 ± 10.1	^b^	88.7 ± 21.3	^b^	86.8 ± 8.4	^b^	97.3 ± 18.8	^b^	104.5 ± 5.6	^b^	125.6 ± 2.2	^b^	213.9 ± 10.5	^a^	<0.0001
mg Omega-3	138.7 ± 15.8		192.1 ± 41.0		163.5 ± 17.6		195.0 ± 34.9		176.6 ± 7.0		170.2 ± 3.1		270.6 ± 11.1		0.0557
mg Omega-6	630.3 ± 18.5	^a,b^	495.8 ± 61.5	^b,c^	355.5 ± 26.0	^c^	402.4 ± 32.4	^c^	683.8 ± 5.9	^a^	381.5 ± 5.4	^c^	395.6 ± 2.1	^c^	<0.0001
mg Omega-6:3	4.54 ± 0.64	^a^	2.58 ± 1.05	^a,b^	2.18 ± 0.17	^a,b^	2.06 ± 0.61	^a,b^	3.87 ± 0.16	^a,b^	2.23 ± 0.01	^a,b^	1.46 ± 0.06	^b^	0.0122
LC-PUFAs	90.5 ± 15.3	^b^	148.5 ± 42.2	^a,b^	133.6 ± 14.2	^a,b^	158.1 ± 34.9	^a,b^	126.0 ± 6.6	^a,b^	140.5 ± 2.5	^a,b^	237.6 ± 11.0	^a^	0.0298
**Rib meat**															
mg ALA	52.8 ± 1.5	^a,b^	50.0 ± 2.5	^b^	66.0 ± 1.2	^a^	65.0 ± 4.8	^a^	41.3 ± 1.4	^b^	47.0 ± 2.4	^b^	26.5 ± 1.7	^c^	<0.0001
mg EPA	12.2 ± 1.4	^a,b^	34.3 ± 13.1	^a,b^	32.8 ± 4.6	^a,b^	46.4 ± 12.4	^a^	8.5 ± 1.0	^b^	10.8 ± 0.6	^a,b^	10.1 ± 0.7	^a,b^	0.0145
mg DPA	35.6 ± 4.4		87.4 ± 31.1		94.8 ± 11.9		100.1 ± 22.2		22.4 ± 3.3		33.0 ± 2.1		25.8 ± 2.1		0.0584
mg DHA	85.3 ± 15.1	^c^	151.6 ± 45.7	^a,b,c^	153.4 ± 18.4	^a,b,c^	186.8 ± 39.1	^a,b,c^	111.2 ± 20.1	^b,c^	260.4 ± 22.2	^a,b^	270.9 ± 31.4	^a^	0.0056
mg Omega-3	231.3 ± 20.6		357.7 ± 89.3		381.2 ± 34.2		431.6 ± 67.8		216.3 ± 23.7		383.2 ± 26.1		349.2 ± 30.9		0.0956
mg Omega-6	1274.7 ± 51.7	^a^	943.9 ± 94.5	^c^	1234.1 ± 28.3	^a,b^	974.3 ± 82.3	^b,c^	993.4 ± 33.5	^a,b,c^	1003.4 ± 19.2	^a,b,c^	518.4 ± 6.1	^d^	<0.0001
mg Omega-6:3	5.51 ± 1.72	^a^	2.64 ± 1.23	^a,b^	3.24 ± 0.42	^a,b^	2.26 ± 0.62	^a,b^	4.59 ± 0.48	^a,b^	2.62 ± 0.15	^a,b^	1.48 ± 0.16	^b^	0.0107
LC-PUFAs	133.1 ± 20.8		273.3 ± 89.2		281.0 ± 34.4		333.3 ± 73.3		142.2 ± 24.0		304.1 ± 23.8		306.7 ± 32.1		0.1059

All values are presented on a per 4oz (113 gram) serving basis.

Means with standard errors followed by different letters are significantly different (*P*<0.05).

At eight weeks, healthy fats were improved significantly for fish fed FO and AM diets (Table 8). More specifically, significant differences (P<0.05) were observed for ALA, DHA, DPA, n-6, n-6:n-3, and LC-PUFAs in the fillet and rib meat of the fish fed different diets. The best diet of the fish oil diets was FO5%. Fish fed FO5% resulted in a n-3 and LC-PUFAs increase of 165 and 232% in the fillet compared to the control. Meanwhile, n-6 and n-6:n-3 ratio were decreased by 2 and 62%. The best diet, AM8.77%, resulted in an increase of n-3 and LC-PUFAs increase of 189 and 298% in the fillet compared to control fed fish. With a corresponding decrease in n-6 and n-6:n-3 by 28 and 75%, respectively. Similar results were observed for the rib meat. Rib meat contained 87% more crude fat compared to the fillet at eight weeks.

**Table 8:** Nutritional fatty acid profiles of tilapia fillet and rib meat after 8 weeks of dietary treatments.

	Control		Fish oil						Algae meal						
		^ ^	1%	^ ^	3%	^ ^	5%	^ ^	1.75%	^ ^	5.26%	^ ^	8.77%	^ ^	P
**Fillet**															
mg ALA	25.9 ± 0.8	^c,d^	33.1 ± 1.5	^a,b^	29.8 ± 0.7	^b,c^	37.1 ± 1.3	^a^	27.8 ± 0.08	^c,d^	25.3 ± 0.6	^c,d^	24.5 ± 0.5	^d^	<0.0001
mg EPA	7.0 ± 1.6		32.1 ± 13.5		26.5 ± 0.8		36.7 ± 11.0		6.2 ± 0.5		7.2 ± 0.4		9.2 ± 0.7		0.0558
mg DPA	22.4 ± 5.3	^b^	71.4 ± 25.4	^a,b^	70.0 ± 1.6	^a,b^	89.5 ± 20.6	^a^	19.4 ± 0.3	^b^	19.1 ± 1.0	^b^	23.1 ± 1.2	^a,b^	0.0049
mg DHA	70.6 ± 12.1	^c^	156.7 ± 44.2	^b,c^	159.6 ± 4.4	^b,c^	205.6 ± 29.8	^b^	162.2 ± 6.7	^b,c^	252.3 ± 3.5	^b^	366.4 ± 12.0	^a,b^	<0.0001
mg Omega-3	151.2 ± 19.0	^b^	320.9 ± 80.4	^a,b^	307.6 ± 6.7	^a,b^	399.4 ± 59.1	^a^	248.9 ± 6.7	^a,b^	324.2 ± 4.5	^a,b^	438.7 ± 14.2	^a^	0.0048
mg Omega-6	784.1 ± 45.0	^a,b^	747.7 ± 115.4	^a,b^	633.4 ± 4.1	^a,b^	770.8 ± 81.3	^a,b^	881.4 ± 12.5	^a^	662.5 ± 8.3	^a,b^	566.6 ± 12.0	^b^	0.0436
mg Omega-6:3	5.19 ± 0.76	^a^	2.33 ± 1.27	^a,b^	2.06 ± 0.03	^b^	1.93 ± 0.55	^b^	3.54 ± 0.08	^a,b^	2.04 ± 0.02	^b^	1.29 ± 0.03	^b^	0.0056
LC-PUFAs	100.0 ± 18.1	^b^	260.1 ± 83.0	^a,b^	255.0 ± 5.5	^a,b^	331.7 ± 60.6	^a^	187.8 ± 7.3	^a,b^	278.6 ± 3.6	^a,b^	398.7 ± 13.6	^a^	0.0052
**Rib meat**															
mg ALA	53.6 ± 4.0		55.7 ± 4.4		56.5 ± 4.1		54.7 ± 7.3		52.7 ± 2.8		52.8 ± 1.3		55.7 ± 15.0		0.9997
mg EPA	13.0 ± 3.4		46.0 ± 20.0		32.4 ± 3.2		45.0 ± 16.4		10.0 ± 1.1		11.3 ± 0.3		14.1 ± 4.0		0.1202
mg DPA	35.7 ± 5.1		95.0 ± 37.3		85.5 ± 7.0		109.4 ± 37.4		31.7 ± 1.8		33.7 ± 1.3		39.1 ± 8.2		0.1071
mg DHA	263.3 ± 159.7		168.7 ± 57.8		143.3 ± 7.4		201.3 ± 44.0		172.1 ± 13.6		383.8 ± 24.5		357.8 ± 77.8		0.3039
mg Omega-3	411.4 ± 160.8		402.5 ± 111.0		351.1 ± 10.8		445.9 ± 99.5		313.8 ± 19.0		515.1 ± 29.2		490.9 ± 47.4		0.7810
mg Omega-6	1496.9 ± 132.8	^a^	1133.1 ± 166.0	^a,b^	1030.7 ± 42.6	^a,b^	1154.3 ± 174.0	^a,b^	1375.3 ± 57.4	^a,b^	1326.8 ± 24.7	^a,b^	848.7 ± 35.4	^b^	0.0242
mg Omega-6:3	3.64 ± 1.54		2.82 ± 1.67		2.94 ± 0.18		2.59 ± 0.99		4.38 ± 0.41		2.58 ± 0.10		1.73 ± 0.31		0.2157
LC-PUFAs	311.9 ± 167.1		309.7 ± 114.4		260.7 ± 17.5		355.7 ± 97.3		213.7 ± 16.2		428.7 ± 26.1		411.0 ± 65.8		0.7470

All values are presented on a per 4oz (113 gram) serving basis.

Means with standard errors followed by different letters are significantly different (*P*<0.05).

Fillet meat increased significantly (P<0.01) from an average of 1.85% to 2.64% in crude fat content from four to eight weeks. Similarly, rib meat increased significantly (P<0.01) from 3.92 to 4.93% crude fat over the same period of time. Healthy fats experienced a similar trend.

Results for liver and mesenteric fat fatty acid profiles are presented in Table 9. Fatty acid profiles of the liver were similar regardless of dietary treatment. Mesenteric fat was similar between the control and FO fed fish. The dose of fish oil did not correlate positively or negatively with the level of fish oil in the diet. However, the fatty acid profile of mesenteric fat correlated with amount if AM in the diet.

**Table 9:** Nutritional fatty acid profiles of tilapia liver and mesenteric fat after 8 weeks of dietary treatments.

	Control		Fish oil						Algae meal						
		^ ^	1%	^ ^	3%	^ ^	5%	^ ^	1.75%	^ ^	5.26%	^ ^	8.77%	^ ^	P
**Liver**															
mg ALA	12.2 ± 0.3		20.2 ± 3.1		22.7 ± 3.3		18.4 ± 2.7		15.7 ± 3.3		14.4 ± 0.6		14.9 ± 2.3		0.2036
mg EPA	6.0 ± 1.0		22.0 ± 9.0		17.6 ± 2.1		23.5 ± 6.9		6.1 ± 0.8		5.7 ± 0.6		7.6 ± 0.6		0.0527
mg DPA	29.3 ± 4.0	^a,b^	51.0 ± 17.8	^a,b^	48.6 ± 9.0	^a,b^	72.9 ± 10.7	^a^	16.1 ± 1.7	^b^	15.5 ± 1.4	^b^	19.6 ± 2.4	^b^	0.0036
mg DHA	287.3 ± 39.0		266.1 ± 86.3		392.7 ± 34.8		511.2 ± 81.5		255.7 ± 36.8		392.9 ± 43.7		434.8 ± 46.0		0.0852
mg Omega-3	368.1 ± 43.6		383.1 ± 109.4		515.5 ± 44.8		667.2 ± 85.1		321.1 ± 44.4		455.9 ± 46.6		494.8 ± 44.8		0.0620
mg Omega-6	888.3 ± 41.1		662.0 ± 81.7		826.2 ± 77.3		941.7 ± 170.9		797.9 ± 92.0		695.5 ± 26.2		518.1 ± 19.3		0.0921
mg Omega-6:3	2.41 ± 0.41		1.73 ± 1.03		1.60 ± 0.12		1.41 ± 0.36		2.49 ± 0.11		1.53 ± 0.11		1.05 ± 0.08		0.1552
LC-PUFAs	322.6 ± 43.4		339.1 ± 110.8		458.8 ± 43.5		607.6 ± 88.0		277.9 ± 41.0		414.1 ± 45.2		462.0 ± 44.6		0.0782
**Mesenteric fat**															
mg ALA	669.4 ± 51.6	^a^	394.1 ± 86.5	^b^	565.6 ± 18.9	^a,b^	438.4 ± 17.6	^a,b^	450.0 ± 22.8	^a,b^	360.6 ± 10.8	^b^	305.8 ± 76.3	^b^	0.0034
mg EPA	133.9 ± 35.3		362.9 ± 138.2		349.1 ± 14.8		328.0 ± 95.3		109.4 ± 20.7		84.2 ± 11.1		112.0 ± 11.4		0.0535
mg DPA	383.7 ± 104.5		855.0 ± 317.0		956.8 ± 39.8		771.4 ± 198.3		276.5 ± 33.8		259.9 ± 22.9		340.8 ± 23.4		0.0568
mg DHA	593.2 ± 143.8	^c^	1041.6 ± 363.8	^b,c^	1280.3 ± 66.5	^b,c^	1002.4 ± 238.6	^b,c^	886.3 ± 57.5	^b,c^	1815.1 ± 34.0	^b^	3306.5 ± 109.2	^a^	<0.0001
mg Omega-3	2297.5 ± 342.0	^a,b^	2934.8 ± 779.9	^a,b^	3465.0 ± 147.3	^a,b^	2771.9 ± 514.6	^a,b^	2028.9 ± 153.5	^b^	2749.4 ± 39.6	^a,b^	4247.0 ± 159.9	^a^	0.0415
mg Omega-6	14886 ± 642.5	^a^	9192.9 ± 962.2	^b,c,d^	10679 ± 603.9	^b,c^	7314.3 ± 746.0	^d^	10870 ± 378.6	^b^	7708.8 ± 102.3	^c,d^	6467.6 ± 125.9	^d^	<0.0001
mg Omega-6:3	6.48 ± 1.27	^a^	3.13 ± 1.59	^a,b^	3.08 ± 0.12	^a,b^	2.64 ± 0.88	^a,b^	5.36 ± 0.31	^a,b^	2.80 ± 0.04	^a,b^	1.52 ± 0.04	^b^	0.0097
LC-PUFAs	1110.8 ± 283.1	^b^	2259.4 ± 765.3	^a,b^	2586.2 ± 98.3	^a,b^	2101.8 ± 531.5	^a,b^	1272.2 ± 113.2	^b^	2159.3 ± 26.4	^a,b^	3759.3 ± 134.4	^a^	0.0247

All values are presented on a per 4oz (113 gram) serving basis.

Means with standard errors followed by different letters are significantly different (*P*<0.05).

## Discussion

Fish demonstrated excellent growth and performance throughout the 8-week feeding trial. Survival ranged from 98%-100%, indicating that fish health was not compromised. Meanwhile, the mean growth rate of fish in this study was good at 45.4±1.0 g a week. Even though other nutritional factors can contribute to changes in deposition of specific fatty acids into different tissues, the treatment diets in our study were consistent across treatment groups.

Numerous efforts have been made to try to increase the n-3 content of tilapia fillets. Addition of flaxseed oil, which is rich in alpha linolenic acid (18:3 n-3), has been found to moderately increase concentration of ALA in tilapia fillets. However, this approach did little to increase LC-PUFAs [11, 12, 20, 21]. This is likely due to that fact that tilapia are limited in their ability to elongate and desaturate (18:3 n-3 and18:3 n-6) into longer chain polyunsaturated fatty acids (20:4 n-6, 20:5 n-3, 22:5 n-3, 22:6 n-3) [22, 23]. The limited ability to synthesize long- chain polyunsaturated fatty acids is also the case for humans. Consequently, tilapia rich in 18:3 n-3 are of little nutritional benefit to consumers [24]. Other vegetable oil replacements, including palm oil and sunflower oil, have resulted in similar beneficial n-3 deficits [25, 26].

From a fish health perspective it has been demonstrated that *Schizochytrium* sp. meal is a suitable replacement ingredient for fish meal and fish oil [27, 28] in tilapia diets. Watters, Rosner [29] determined that *Schizochytrium* sp. and fish oil can boost n-3 fatty acids in tilapia fillets over a period of six months. In contrast, tilapia in this study achieved similar increased n-3 values within four week. Meanwhile, Sarker et al. [13, 23] demonstrated that *Schizochytrium* sp. in diets improved growth and fatty profiles in juvenile tilapia. The Sarker et al. [13, 23] studies also demonstrated that increased n-3 can be achieved in the fillet of fish in a short period of time for young tilapia that were cultured up to 25 grams. In the study herein, tilapia were grown up to market size and tissues other than the fillet were characterized for increased nutritional profiles.

In the wild, tilapia fatty acid composition fluctuates with location and season [30, 31] However in controlled RAS systems, other factors affect fatty acid metabolism including feeding frequency, starvation, and water temperature [32, 33]. All of these conditions factor into how tilapia utilize dietary fatty acids and proteins as energy sources. The colder the water temperature, the more efficient fish are at converting saturated fatty acids into monounsaturated and polyunsaturated fatty acids [33, 34]. This is possibly due to the need to keep cell membranes fluid at lower temperatures, and polyunsaturated fatty acids provide greater membrane fluidity. Since tilapia were kept ~29°C throughout this study, it is likely that this moderate temperature did not inhibit the desaturation and elongation of saturated fatty acids to mono- and polyunsaturated fatty acids. Starvation resulted in utilization of fatty acids in the liver as an energy source as opposed to muscle fatty acids [32]. Because fish in this study were not starved prior to sampling, livers were observed to be high in fat (Table 9).

Fish store lipids in a variety of tissues including fillet (dark muscle), rib meat (light muscle), liver, and mesenteric fat. Each of these tissues provides a different function for lipid storage and processing. Mesenteric fat typically provides long-term storage of lipids, liver performs lipid processing, and light and dark muscle functions as more short-term storage for localized energy requirements [35]. Tilapia tend to have more fat stored in liver compared to muscle, on a percent weight basis [36]. The results of this study agree with this, every diet resulted in a greater percent lipid in liver compared to fillet.

Other tissues that would normally be considered byproducts including rib meat, mesenteric fat and liver could also be developed into value-added products. Rib meat could be formulated into sausages, surimi, or salt-biscuits as all of these products have been created with positive sensory characteristics [37–39]. Liver and mesenteric fat tissues could also be incorporated into a high n-3 pet food [40]. At 8 weeks, the liver tissue of tilapia fed experimental fish oil and AM diets had a similar composition of LC-PUFAs to fillets of tilapia fed the same diets at the same time, between~100–200 mg (Tables 5 and 6). Szabo, Mezes [41] found when tilapia were fed various vegetable oils, long-chain polyunsaturated fatty acids would accumulate in the liver as opposed to the fillet. This study demonstrates that it is possible for tilapia to store LC-PUFAs in both liver and fillet tissues. In addition, at 8 weeks the mesenteric fat was saturated with LC-PUFAs with 1362 mg per serving in fish fed 5% fish oil and 1504 mg per serving in fish fed 8.77% AM (Table 7). In general, the lowest omega-6:3 ratio was present in the liver and the highest in mesenteric fat. This suggests that more omega-6 fatty acids was partitioned for long-term storage compared to omega-3. This could also indicate a preference to utilize omega-3 fatty acids as short-term energy sources compared to omega-6 fatty acids.

Beneficial n-3 composition is observed to increase linearly with percent AM at 4 weeks with a line equation of y = 8.8525x + 63.657 and an R^2^ = 0.9946, calculated from Table 7. The same trend is observed at 8 weeks with a line equation of y = 14.973x + 78.091 and an R^2^ = 0.9885, calculated from Table 8. This indicates that percent AM in the diet and beneficial n-3 content in the fillet are strongly positively correlated. This was also indicative that tilapia fed increasing percent AM diet do not readily utilize the LC-PUFAs themselves, but, instead store them. This is possibly due to the high protein content of the feed, between 35.5–37.3%. As tilapia grow, their relative protein requirement decreases and the required digestible energy can be replaced with carbohydrate [42]. It is recommended that commercial tilapia feeds for fry are typically between 35 and 40% protein and for fingerlings to harvest size between 32–35%. This is true for both outdoor pond cultivation and RAS cultivation; however, the quality and purity of protein used in RAS systems is generally higher than in pond production because in pond production fish can supplement feed with environmental protein sources. Because these diets were so high in energy from protein sources relative to the fishes’ nutritional requirement, the majority of high energy polyunsaturated fatty acids including LC-PUFAs were able to be stored in tissues instead of being utilized as an energy source.

Fish fed either the 8.77% AM, or the 5% fish oil diets resulted in >200mg DHA per 4oz (113 g) serving. This is more than commercially available channel catfish, Atlantic and Pacific cod (137mg, 154mg, and 173mg respectively) (USDA, 2005). This demonstrates that farmed tilapia fed these diets show a nutritional improvement over other low fat white fish. Future research would include the economic feasibility of a high percent AM diet compared to the added value to consumers of n-3 enriched tilapia fillets. This would solidify the use of practical alternatives to fish oil as a method of modifying n-3 content of tilapia fillets. New advancements in the production of *Schizochytrium* sp. could lead to the rapid, sustainable, and economical cultivation of DHA-rich microalgae [43, 44]. Also observing if the linear trend of beneficial n-3 fillet content continues with increasing percent of AM beyond 8.77% should be pursued. If the trend continues, it may be possible to develop a finishing feed with very high AM (i.e. possibly 10% of diet) that deposits the desired quantity of beneficial n-3 into the fillet quicker and therefore more cost-effectively.

## Conclusions

Overall the experimental diets presented in this study show promise as a feasible option for enriching beneficial n-3 content in tilapia fillets. Tilapia in this study also demonstrated the ability to elongate and desaturate shorter chain polyunsaturated fatty acids into longer chain polyunsaturated fatty acids. The continuous feeding along with moderate temperatures, high protein and high-n-3 diets resulted in rapid fish growth and beneficial n-3 enriched fillets. This study also suggests that tilapia fed these diets could produce value-added byproducts, by using n-3 enriched rib meat, liver and mesenteric fat tissues in other processed foods.

## Supporting information

S1 Table Fat content (g per 4 oz. [113 g] serving) in various tissues of fish fed different experimental diets. (XLSX)
